# Spines, Plasticity, and Cognition in Alzheimer's Model Mice

**DOI:** 10.1155/2012/319836

**Published:** 2011-11-28

**Authors:** Tara Spires-Jones, Shira Knafo

**Affiliations:** ^1^Massachusetts General Hospital, Harvard Medical School, 114 16th Street, Charlestown, MA 02129, USA; ^2^Centro de Biología Molecular “Severo Ochoa,” Consejo Superior de Investigaciones Científicas (CSIC) and Universidad Autónoma de Madrid, Nicolás Cabrera, 28049 Madrid, Spain

## Abstract

The pathological hallmarks of Alzheimer's disease (AD)—widespread synaptic and neuronal loss and the pathological accumulation of amyloid-beta peptide (A**β**) in senile plaques, as well as hyperphosphorylated tau in neurofibrillary tangles—have been known for many decades, but the links between AD pathology and dementia and effective therapeutic strategies remain elusive. Transgenic mice have been developed based on rare familial forms of AD and frontotemporal dementia, allowing investigators to test in detail the structural, functional, and behavioral consequences of AD-associated pathology. Here, we review work on transgenic AD models that investigate the degeneration of dendritic spine structure, synaptic function, and cognition. Together, these data support a model of AD pathogenesis in which soluble A**β** initiates synaptic dysfunction and loss, as well as pathological changes in tau, which contribute to both synaptic and neuronal loss. These changes in synapse structure and function as well as frank synapse and neuronal loss contribute to the neural system dysfunction which causes cognitive deficits. Understanding the underpinnings of dementia in AD will be essential to develop and evaluate therapeutic approaches for this widespread and devastating disease.

## 1. Introduction

Alzheimer's disease (AD) is a devastating progressive neurodegenerative disease characterized by cognitive decline, brain atrophy due to neuronal and synapse loss, and the formation of two pathological lesions, extracellular amyloid plaques composed largely of amyloid-beta peptide (A*β*), and neurofibrillary tangles, intracellular aggregates of hyperphosphorylated tau protein [1, 2].

The brain is a remarkably adaptable network of neurons sharing information through approximately 10^14^ synaptic connections. The plasticity of this network in response to environmental stimuli enables the brain to adapt to new demands and allows learning and the formation of new memories. Changes of synapses and dendritic spines, the postsynaptic element of most excitatory synapses, in response to stimulation are thought to underlie the brain's plasticity [3]. It follows that disruption of neural circuits due to both synapse loss and decline in the ability of remaining spine synapses to change in response to stimuli likely contribute to the disruption of cognition observed in neurodegenerative diseases such as Alzheimer's disease. Indeed, it is known that synapses are lost during AD and that, in AD tissue, synapse loss correlates strongly with cognitive decline, arguing the importance of this process as causative to dementia [4–7].

 Rare familial forms of AD occur in which amyloid precursor protein (APP), the precursor to the A*β* peptide, or presenilin (PS) 1 or 2, the catalytic subunit of the gamma-secretase complex which cleaves APP to form A*β*, are mutated and result in an autosomal dominant, early-onset form of the disease [8]. Mutations in the tau protein have not been found to cause AD but can lead to familial frontotemporal dementia with Parkinsonism linked to chromosome 17 [9, 10]. These mutations strongly implicate amyloid processing as an instigating factor in the disease and also provide genetic tools for the construction of transgenic mouse models of the disease which recapitulate many of its pathological features [11]. In contemporary AD research, these transgenic models are being used to characterize the physiological and behavioral consequences of AD neuropathology in order to investigate the fundamental question of the underlying anatomical causes of dementia. APP and APP/PS1 transgenic mice express high levels of amyloid beta (A*β*) and progressively develop many of the pathological phenotypes of AD, including abundant extracellular A*β* plaques, synaptic dysfunction and loss, astrocytosis, activation of microglia, and cognitive deficits [12]. For decades, A*β* plaques were thought to cause dementia in AD patients by physically interrupting normal neural connectivity and function. However, the lack of correlation between A*β* plaque load and the degree of cognitive impairment in AD patients [4] and the fact that A*β* plaques occupy a negligible fraction (less than 5%) of the neuropil [13–15] in cognitively impaired transgenic mice [15] raised the possibility that fibrillar A*β* in plaques does not contribute significantly to dementia in AD patients. Instead, soluble A*β* species (i.e., monomeric, oligomeric, and protofibrillary A*β* species that linger in aqueous solution after high-speed centrifugation) seem to be the main culprits of the functional deficits in these mice and probably also in initiating disease in AD patients.

Mice expressing dementia-associated tau mutations have also been developed to study the contributions of neurofibrillary pathology to dementia and the interplay between A*β* and tau [16, 17]. While genetic studies clearly implicate amyloid as the initiating factor in AD, the correlation of tangles with neuronal loss in AD brain, together with the lack of neuronal loss and tangle formation in APP transgenic models, and the lack of efficacy with A*β*-directed therapeutics have contributed to the idea that tau pathology is an important contributor to dementia downstream of A*β* [18].

This paper will review the work on dendritic spine changes and their contribution to functional changes in synapses and behavioral deficits in AD models. It is important to address these questions because the ability of synapses and spines to change even in aged brain and the strong correlation between synapse loss and cognitive decline in AD indicate that enhancing spine plasticity could prevent or even reverse cognitive deficits associated with neurodegenerative disease.

## 2. *In Vivo* Imaging Reveals Dendritic Spine Loss and Plaque-Associated Structural Plasticity Deficits in AD Models

Dendritic spines form the postsynaptic element of the vast majority of excitatory synapses in the cortex and hippocampus brain regions important for learning and memory. Changes in spines are thought to be a structural basis for these processes [3]. Loss of dendritic spines similar to the synapse loss observed in human AD has been reported in several mouse models that develop amyloid and tau pathology [13, 19–25]. The use of mouse models for *in vivo *multiphoton imaging allows longitudinal investigations to determine the temporal sequence of pathological events and to answer “chicken-or-egg” questions such as which comes first, spine loss or plaques? In order to perform these experiments, it is first necessary to fluorescently label dendritic spines and pathological lesions such as plaques and tangles. Spines can be imaged with transgenic expression of fluorophores such as GFP and YFP [26–29] or through filling neurons with fluorescent dextrans [30] or fluorescent proteins expressed in adeno-associated virus or lentivirus [21, 31]. To label plaque pathology in AD models, the blood-brain barrier-penetrable compounds, Pittsburghs compound B and methoxy-XO4 (developed by William Klunk), have been used in conjunction with *in vivo *multiphoton imaging to observe amyloid plaques and their clearance after treatment with immunotherapy [32–35].

**Figure 1:**
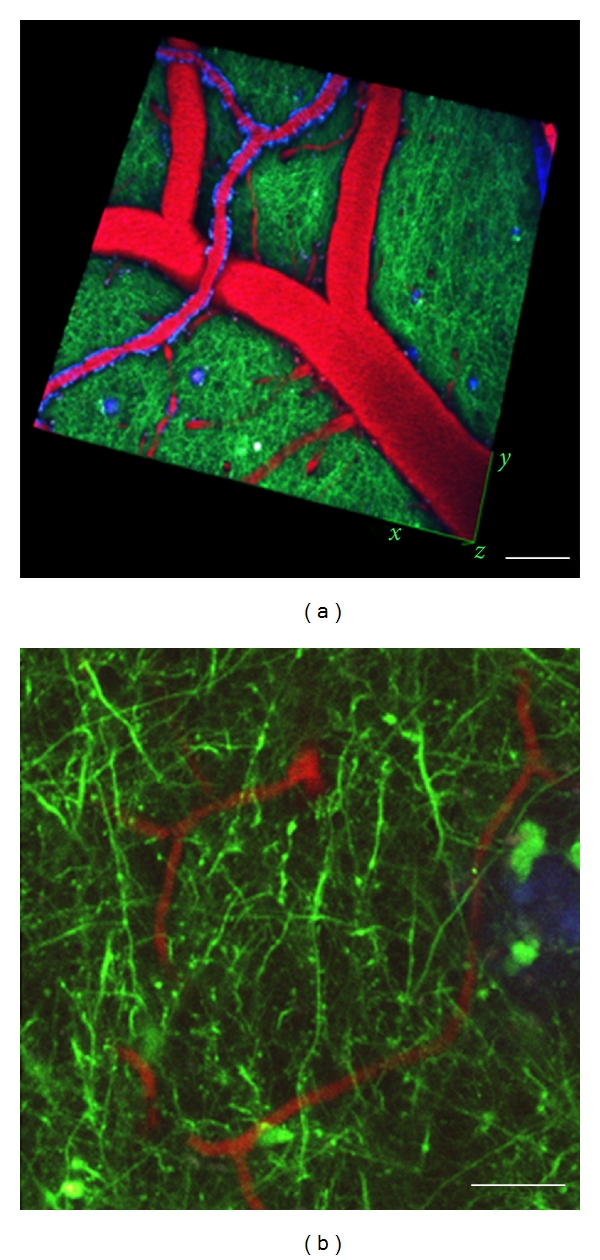
*In vivo *multiphoton imaging of plaques (labeled with methoxy X-O4, blue), vasculature (labeled with Texas red dextran, red), and dendrites (transgenically expressing YFP, green) in mice transgenic for mutant human APP and PS1 crossed with YFP transgenic mice allow examination of dendritic spine plasticity and loss. Low-resolution three-dimensional image stacks (a) are used to repeatedly find the same imaging sites. Higher-resolution image stacks (b) are used for spine analysis. Scale bars 100 *μ*m (a) 10 *μ*m (b).

Imaging of amyloid plaques together with imaging dendrites and dendritic spines filled with fluorescent proteins can be used to assess the effects of pathology on the surrounding neuropil (Figure 1). This technique shows that plaques form rapidly, over the course of one day, and that within one week of plaque formation, surrounding dendrites begin to curve and exhibit dystrophic swellings [36]. Spine loss around plaques was determined to be due to a loss of stability of spines in the vicinity of plaques with more spine elimination than that in control brain, reflecting dysfunctional structural plasticity [37]. These structural plasticity changes contribute to functional deficits around plaques. In one study, neural circuit function was assessed using a fluorescent reporter of neuronal activation (the coding sequence of Venus, flanked by short stretches of the 5′ and 3′ untranslated regions from CamKII*α*) which gets transported to dendrites and locally translated in response to activity resulting in increased fluorescence in dendrites after neuronal activation. APP/PS1 transgenic mice have greatly reduced levels of this reporter in dendritic segments surrounding plaques, and they failed to upregulate its expression in response to environmental stimulation, a phenomenon which was robust in wild-type animals [38]. Resting intraneuronal calcium levels are also disrupted around plaques, indicating dysfunction [39].

From the above studies, it is clear that plaques affect local dendrites and dendritic spines, but the precise bioactive molecule around plaques which induces spine loss was not clear for many years. The strongest candidate for the synaptotoxic molecule around plaques arose as soluble oligomeric A*β* due to work by William Klein, Dennis Selkoe, and other groups who reported that soluble A*β* causes dendritic spine collapse and impairs synaptic plasticity in culture [40–44], correlates with memory loss in transgenic mice [45, 46], and impairs memory and synaptic plasticity *in vivo *[47, 48]. In further support of the synaptotoxic role of A*β*, both active and passive immunotherapy to remove A*β* have favorable effects on memory, plaque clearance, and neurite architecture in AD models [33, 49–53]. The first direct assessment of whether oligomeric A*β* is present at synapses in the brain came from application of the array tomography technique to AD mouse brain tissue. Array tomography, developed by Micheva et al., overcomes the axial resolution limitation of confocal microscopy by physically sectioning tissue into 70 nm ribbons of serial sections which can then be used for immunofluorescent analysis to accurately quantify the contents of small structures such as synapses [54, 55]. In APP/PS1 mice, this technique shows that oligomeric A*β* is in fact present at a subset of shrunken excitatory postsynaptic densities, particularly in a halo of oligomeric A*β* surrounding the dense cores of plaques [56]. As would be predicted from the association of dendritic spine changes with physiological plasticity [57–59] and the presence of oligomeric A*β* at shrinking spines, dendritic spines can recover with therapeutic interventions aimed at removing oligomeric A*β* or inhibiting calcineurin which is activated downstream of A*β*-associated increases in intracellular calcium [30, 39, 60–62]. Removing soluble A*β* with the topical application of the 3D6 antibody allows rapid increases in the structural plasticity of dendritic spines within one hour, before any clearance of fibrillar A*β* occurs [30].

Tau overexpression has also been associated with spine loss in postmortem studies of human tau transgenic animals [25]. In rTg4510 mice, pyramidal cells have reduced spine density compared to wild-type animals, but tangle-bearing neurons have no more loss than their non-tangle-bearing neighbors [25]. Similarly rTg4510 hippocampal circuits are deficient in experience-dependent upregulation of immediate early genes compared to wild-type mice, but tangle-bearing neurons are not impaired compared to non-tangle-bearing cells in rTg4510 brain [63]. *In vivo* and array tomography imaging of tangles in rTg4510 mice has been developed and is demonstrating similar indications that soluble tau may be more toxic than fibrillar tau in terms of axonal transport and neuronal death [64–68]. In cultured neurons and transgenic mice overexpressing tau, mislocalization of tau to dendritic spines disrupts synaptic function [69].

Very recent data elegantly link A*β*, tau, and dendritic spine loss [70, 71]. Ittner et al. established that tau has a dendritic function in targeting the Src kinase Fyn to dendritic spines. Fyn phosphorylates NMDA receptor NR2 subunits mediating their interaction with the postsynaptic scaffolding protein PSD95 and disrupting this interaction of tau, and Fyn prevents A*β* toxicity in APP transgenic mice [70]. Similarly, Roberson et al. found that A*β*, tau, and Fyn jointly impair synaptic network function in electrophysiological studies of APP and Fyn overexpressing mice on a tau null background [71]. In culture, oligomeric A*β* was found to cause tau mislocalization to dendrites which was associated with local calcium elevation and dendritic spine loss [72].

## 3. Synaptic Plasticity Is Severely Impaired in AD Mouse Models

It is widely accepted that, in early stages of AD, synaptic dysfunction is the cause of dementia [84, 85]. Synaptic plasticity provides a neurophysiological substrate for learning and memory and is, therefore, often used to evaluate the phenotype of transgenic mice. In APP transgenic AD mouse models, there are significant alterations in hippocampal synaptic transmission and plasticity at excitatory glutamatergic synapses that sometimes appear in young animals long before A*β* is deposited in plaques (see Table 1). Most studies performed before mice reached 6 months of age report intact basal synaptic transmission [75, 78, 81, 83] although some exceptions were also reported [74, 86]. It should be noted that the lack of detectable changes in basal synaptic transmission in the majority of studies does not rule out synaptic dysfunction that has been overcome by functional compensation. Indeed, evidence of functional compensation in response to spine loss induced by A*β* has been observed in several models [87–89]. From 6 months on, most of the AD transgenic mice show significant deficits in basal synaptic transmission [75, 78, 79, 81–83, 90]. This age-related deterioration in synaptic transmission in AD transgenic mice is unlikely to result from a decreased transmitter release probability because paired-pulse facilitation (PPF), which correlates inversely with the probability of transmitter release [78], remains intact in most of AD transgenic mice, even at advanced ages [78, 86] (see Table 1). Impairments in long-term potentiation (LTP) were shown both *in vitro* and *in vivo*, in the CA1 as well as dentate gyrus regions of the hippocampus [76, 91]. Failure of LTP expression is detected in AD mice in some cases before 4 months of age [73, 78] but usually appears later [75, 76, 81–83, 86, 92], when A*β* load is higher. These findings emphasize the fact that extracellular deposition of fibrillar A*β* is not required for the development of severe functional deficits in AD models. This conclusion is strengthened by the observation that direct application of A*β* oligomers into the brain prevents LTP [48, 93, 94].

Studies of the mechanisms of A*β*-mediated synaptic dysfunction converge on the theme of increased postsynaptic calcium concentrations leading to internalization of NMDA and AMPA receptors via mechanisms similar to those seen in long-term depression [40, 42, 95, 96]. Overall, these findings suggest that synaptic dysfunction is an early event in AD pathogenesis and may play a role in the disease process.

## 4. Impaired Cognition in AD Mouse Models

Learning and memory processes are believed to depend on changes of synaptic transmission in certain areas of the brain, including the hippocampus. Most studies done with AD transgenic mice assess spatial navigation capability (e.g., Morris water maze, radial maze, Barnes maze) since this memory system depends on the hippocampus and is highly conserved in mammals [97]. The onset of cognitive decline is difficult to define in humans, particularly without a reliable biomarker. Thus, the use of transgenic mouse models to address this question is particularly useful, since the early cognitive changes can be identified and correlated with molecular and cellular changes. The implication of A*β* in the cognitive decline in AD transgenic mice is no longer controversial. However, there were contrasting reports regarding the onset of cognitive decline in different AD models (Table 2). In some studies, deficits in learning and memory were observed at 3 months, implicating soluble A*β* assemblies [98, 99], while other studies have shown onsets at intermediate ages [76, 98, 100–104] or at advanced ages [100, 101, 105, 106], invoking insoluble A*β* plaques. Moreover, in the 3XTg-AD mouse model, spatial long-term retention memory deficits were found to correlate with intraneuronal A*β* at 4 months [81], an age when these transgenic mice do not have A*β* plaques [107]. A similar observation has been shown for 5x FAD mice, which also accumulate high amounts of intraneuronal A*β* peptides [108] and present with significant impairment in the working memory already at 4-5 months of age [102, 103]. Due to this controversy, the A*β* species responsible for the cognitive decline in these mice was under debate for many years. Strong evidence for the toxicity of soluble A*β* came from a study showing that naturally secreted soluble A*β* oligomers administrated into the rat's lateral cerebral ventricles at picomolar concentrations disrupt the memory of a complex learned behavior [47]. This suggests that soluble A*β* oligomers, rather than A*β* plaques, may be responsible for the cognitive impairment in the absence of A*β* plaques or neuronal death.

Although aged AD mice are impaired at learning several tasks that depend on the hippocampus, the performance of these mice on tasks requiring an intact amygdala, such as cued-fear conditioning, has been thoroughly established only for Tg2576 mice [112, 119] and aged APP/PS1 mice [15]. In these models amygdala-dependent learning is severely impaired at advanced ages, implying that neurons of the amygdala, similar to hippocampal neurons, are susceptible to the toxic effect of A*β*.

At later stages of the disease, widespread synaptic and neuronal death probably contribute greatly to dementia. These effects are likely mediated by tau downstream of the initiating amyloid pathology [18]. Reflecting this later stage of dementia, tau-expressing mouse lines which undergo neuronal loss develop behavioral deficits. Interestingly, two of these mouse lines which have reversible expression of pathological tau exhibit recovery of cognition after transgene suppression even after extensive neuron loss [147, 148]. These studies point to the powerful ability of synapses to regenerate and allow functional recovery of neural circuits if the toxic insult in the disease can be removed.

## 5. Conclusions

The data presented in this paper are from a strong body of literature supporting the hypothesis that oligomeric A*β* accumulation in the brain initiates the disease process in AD by impairing structural and functional plasticity of synapses. This underlies behavioral deficits observed in APP mouse models which begin before A*β* deposition in plaques and continue after plaque deposition when the plaques appear to be a reservoir of oligomeric A*β* causing local structural and functional disruptions. Downstream of the initial amyloid insult, tau pathology contributes to synapse and neuronal loss and consequent cognitive decline. AD transgenic mice are characterized by a number of specific cognitive deficits, compatible with AD, which makes them indispensable for testing of novel anti-AD drugs. Finally, the plastic nature of synapses and their clear involvement in both early and late stages of cognitive decline in these AD models highlight the importance of synaptic targets for therapeutic approaches.

## Figures and Tables

**Table 1:** Progressive synaptic malfunction AD transgenic mice.

Model	Mutations	Age (months)	Basal synaptic transmission	Long-term potentiation	Paired-pulse facilitation
		2-3		Impaired [73]	
Tg2576	APPswe	4–6	Impaired [74]	Normal [74]/impaired [73]	
		6–12	Impaired [75]		Normal [75]
		>12	Normal [76, 77]/Impaired [75]	Normal [75]/impaired [76, 77]	Normal [4, 6]

		<6	Normal [78]	Impaired [78]	Impaired [78]
PDAPP	APP (V717F)	6–12			
		>12	Impaired [78]	Normal [78]	Impaired [78]

		<6	Normal [79]	Impaired [79, 80]	
APP/PS1	APPswe/PS1dE9	6–12	Impaired [79, 80]	Impaired [79, 80]	Normal [80]
		>12			

		1-2	Normal [81]	Normal [81]	Normal [81]
3xTg-AD	APPSwe, PS1M146V, and tauP301L	3–6			
		6–12	Impaired [81]	Impaired [81]	Normal [81]
		>12			

5XFAD	APP_swe/lnd/fl_ and a PS1 transgene carrying double FAD mutations (M146L and L286V)	<6	Normal [82]	Normal [82]	Normal [82]
		6–12	Impaired [82, 83]	Impaired [82, 83]	Normal [82]
		>12			

**Table 2:** Progressive cognitive impairments in APP AD transgenic mice.

Model	Age (months)	Spatial task		Working memory	Fear conditioning	
		Learning	Probe test		Contextual	Cued
Tg2576	<6	Impaired [109]/normal [98, 100, 110]	Normal [100, 111]	Normal [110]/impaired [98]	Impaired [74, 109, 112–118]	Normal [119]
	6–12	Normal [101]/impaired [110, 120]	Normal [120]/impaired [111]	Normal [98, 101]/impaired [110]	Impaired [115, 121]/normal [122]	Normal [122]
	>12	Impaired [101, 110]	Impaired [111]	Impaired [76, 98, 101, 110]	Impaired [112, 115, 117]	Normal [112, 119]/impaired [119]

APP/PS1	<6	Normal [123–128]	Normal [126, 127]/impaired [128]	Normal [124, 127, 129, 130]/impaired [80]	Normal [131–133]/impaired [79, 117]/enhanced [127]	
	6–12	Impaired [79, 80, 105, 126–128, 134]	Normal [105, 127, 134]/impaired [79, 80, 126, 128]	Impaired [80, 127, 129, 130]	Impaired [79]/normal [127]	
	>12	Impaired [105, 106, 124, 128, 135]	Impaired [105, 106]	Impaired [124, 129]		Impaired [15]

3xTg-AD	1-2	Normal [107]	Normal [107]	Normal [107, 136]		
	3– 6	Impaired [137–139]	Impaired [107, 137, 138]	Normal [136]		
	6–12	Impaired [107, 140]	Impaired [107, 139, 140]	Impaired [136, 139, 141]	Impaired [139]	
	>12		Impaired [142]	Impaired [141, 143]		

5XFAD	<6		Impaired [108, 144]	Normal [102, 108]	Normal [82, 145]	Normal [144]
	6–12	Impaired [144]		Impaired [102, 103]	Impaired [82, 144, 145]	
	>12			Impaired [102, 103, 146]		
